# Supplementary material for: Leaf Aquaporin Expression in Grafted Plants and the Influence of Genotypes and Scion/Rootstock Combinations on Stomatal Behavior in Grapevines Under Water Deficit
Source: Plants (Basel). 2024 Dec 6;13(23):3427. doi: 10.3390/plants13233427 (PMC11644422; doi:10.3390/plants13233427)
Supplement: Supplementary file 1 [file plants-13-03427-s001.zip › plants-3302925-supplementary.pdf]

Supplementary information

Table S1. Environmental conditions during the 21 days' experiments, throughout the day (average) and at noon (time of measurements, between 11 am and 3 pm). Temperature and relative humidity were monitored with a hygrothermograph from a data logger (HOBO® Pro v2, Onset Computer Corporation, Bourne, MA, USA) under the same plant conditions that allowed the vapor pressure deficit (VPD) estimation.

Parameter		Nongrafted genotype experiment (01/23/17-02/12/17)	Scion/rootstock combinations experiment (02/20/17-03/12/17)
Temperature			
	Average	24,1 °C	23,3 °C
	Noon	33,9 °C	33,0 °C
Relative humidity			
	Average	52,07 %	52,78 %
	Noon	30,2 %	31,17 %
Vapor Pressure Deficit			
	Average	1,44 kPa	1,35 kPa
	Noon	3,69 kPa	3,46 kPa

Table S2. Comparative table summarizing the differences in drought tolerance, nutrient uptake, and vigor among the Vitis rootstocks 1103 Paulsen (1103P), SO4, and 101-14, as well as the cultivars Cabernet Sauvignon and Carmenere (Adapted from Keller [11]).

Trait	1103 Paulsen (1103P)	SO4	101-14	Cabernet Sauvignon	Carmenere
**Drought Tolerance**	High1	Moderate2	Low2	Moderate3	Moderate3
**Nutrient Uptake**	High2	High2	Moderate2	Moderate	Moderate
**Vigor**	High2	Moderate2	Low2	High	High

Table S3. Plant height (cm) for irrigation treatments and genotypes analyzed. Each number represents two sample media.

Irrigation treatment	Genotype	Day 1 (cm)	Day 7 (cm)	Day 14 (cm)	Rec (cm)
Ni	1103P	71.5	78.5	80	83
	SO4	72.0	75.5	79	80
	CAR	37.0	42	42.5	45
	CS	52.0	59.5	59.5	61.5
Fi	1103P	68.5	86.5	101.5	118
	SO4	71.0	86.5	107.5	123.5
	CAR	56.5	67	86	99
	CS	43.5	57.5	75.5	91

Table S4. Foliar area (cm^2) for irrigation treatments and genotypes analyzed. Each number represents two sample media.

Irrigation treatment	Genotype	Day 1 (cm^2)	Day 7 (cm^2)	Day 14 (cm^2)	Rec (cm^2)
Ni	1103P	523.09	469.74	364.20	358.39
	SO4	1101.50	801.31	708.98	344.62
	CAR	804.64	727.47	303.16	429.07
	CS	905.69	759.79	490.51	249.28
Fi	1103P	483.58	476.92	489.46	505.30
	SO4	900.05	898.56	1101.88	639.65
	CAR	565.00	656.20	932.74	720.81
	CS	603.39	690.74	749.03	770.16

Table S5. Mean chlorophyll content (SPAD units) for irrigation treatments and genotypes analyzed. Each number represents two sample media.

Irrigation treatment	Genotype	Day 1*	Day 7*	Day 14*	Rec*
Ni	1103P	31.48	29.15	29.45	28.35 B
	SO4	33.60	30.93	29.25	31.15 B
	CAR	35.05	32.45	30.98	33.80 B
	CS	39.03	38.20	37.28	36.75 B
Fi	1103P	33.60	29.85	31.38	33.50 A
	SO4	35.00	31.10	30.55	34.45 A
	CAR	34.60	33.98	32.30	33.80 A
	CS	41.30	37.38	38.23	38.45 A

Rec Differences due to irrigation treatment p=0.042

*Differences due to genotype, independent of water treatment Day 1 (p=0.00085), CS>CAR=SO4=1103P; Day 7 (p=0.0227) CS>CAR=SO4=1103P; Day 14 (p=0.0266) CS=CAR≥1103P=SO4; Rec (p=0.0013).

Table S6. Maximum quantum yield of primary photochemistry (Fv/Fm) for irrigation treatments and genotypes analyzed. Each number represents two sample media. No significant differences were observed.

Irrigation treatment	Genotype	Day 1	Day 7*	Day 14	Rec
Ni	1103P	0.80	0.82	0.80	0.80
	SO4	0.81	0.80	0.80	0.79
	CAR	0.78	0.79	0.72	0.79
	CS	0.79	0.81	0.77	0.78
Fi	1103P	0.79	0.82	0.82	0.81
	SO4	0.80	0.81	0.79	0.79
	CAR	0.80	0.81	0.81	0.80
	CS	0.79	0.82	0.81	0.81

Table S7. Soil moisture content (cm^3/cm^3) for irrigation treatments and genotypes analyzed. Each number represents two sample media.

Irrigation treatment	Genotype	Day 1 (cm^3/cm^3)	Day 7 (cm^3/cm^3)	Day 14 (cm^3/cm^3)	Rec (cm^3/cm^3)
Ni	1103P	0.16	0.11	0.03	0.21
	SO4	0.10	0.02	0.02	0.20
	CAR	0.02	0.04	0.03	0.21
	CS	0.07	0.02	0.02	0.16
Fi	1103P	0.04	0.21	0.16	0.11
	SO4	0.04	0.27	0.24	0.14
	CAR	0.06	0.21	0.22	0.03
	CS	0.08	0.25	0.22	0.13

Table S8. Plant height (cm) for irrigation treatments and scion/rootstock combinations analyzed. Each number represents three sample media.

Irrigation treatment	Combination	Day 1 (cm)	Day 7 (cm)	Day 14 (cm)	Rec (cm)
Ni	CS/CS	49.0	50.3	50.7	51.3
	CS/101-14	77.3	80.3	81.7	83.3
	CS/1103P	58.0	66.0	66.0	66.7
Fi	CS/CS	65.3	66.0	68.0	70.3
	CS/101-14	80.0	87.7	102.0	103.8
	CS/1103P	48.3	53.7	63.7	69.3

Table S9. Foliar area (cm^2) for irrigation treatments and scion/rootstock combinations analyzed. Each number represents three sample media.

Irrigation treatment	Combination	Day 1 (cm^2)	Day 7 (cm^2)	Day 14 (cm^2)	Rec (cm^2)
Ni	CS/CS	669.97	521.62	477.98	340.78
	CS/101-14	789.76	883.37	727.36	829.16
	CS/1103P	537.56	631.41	624.93	714.89
Fi	CS/CS	607.68	650.05	428.57	782.84
	CS/101-14	979.73	1069.12	1282.55	1196.86
	CS/1103P	389.64	528.66	613.84	687.01

Table S10. Mean chlorophyll content (SPAD units) for irrigation treatments and scion/rootstock combinations analyzed. Each number represents three sample media.

Irrigation treatment	Combination	Day 1	Day 7	Day 14*	Rec
Ni	CS/CS	40.77 B	39.67	41.48	37.23
	CS/101-14	38.72 B	37.12	37.05	41.13
	CS/1103P	37.00 B	37.77	37.68	37.07
Fi	CS/CS	38.52 A	39.13	38.50	42.07
	CS/101-14	32.57 A	36.38	39.57	37.53
	CS/1103P	33.20 A	37.85	37.33	38.47

Day 1, differences due to water treatment (p=0.028),

Day 14, differences due to combination (p=0.037) and, irrigation treatment and combination interaction (p=0.026) CS/CS Ni > CS/101-14Fi ≥ CS/CS Fi ≥ CS/1103P Ni > CS/1103P Fi > CS/101-14 Ni

Table S11. Maximum quantum yield of primary photochemistry (Fv/Fm) for irrigation treatments and scion/rootstock combinations analyzed. Each number represents three sample media.

Irrigation treatment	Combination	Day 1	Day 7	Day 14	Rec*
	CS/CS	0.79	0.81		0.79
	CS/101-14	0.79	0.80		0.78
Ni	CS/1103P	0.77	0.81		0.79
Fi	CS/CS	0.80	0.81		0.81
	CS/101-14	0.79	0.79		0.79
	CS/1103P	0.79	0.80		0.79

On Day 14th data could not be registered

*Differences due to combinations (p=0.048) CS/CS ≥ CS/1103P ≥ 101-14

Table S12. Soil moisture content (cm^3/cm^3) for irrigation treatments and scion/rootstock combinations analyzed. Each number represents three sample media.

Irrigation treatment	Combination	Day 1 (cm^3/cm^3)	Day 7 (cm^3/cm^3)	Day 14 (cm^3/cm^3)	Rec (cm^3/cm^3)
	CS/CS	0.24	0.08	0.03	0.23
Ni	CS/101-14	0.20	0.05	0.01	0.16
	CS/1103P	0.20	0.05	0.02	0.14
Fi	CS/CS	0.23	0.21	0.18	0.18
	CS/101-14	0.19	0.20	0.17	0.11
	CS/1103P	0.23	0.22	0.18	0.24

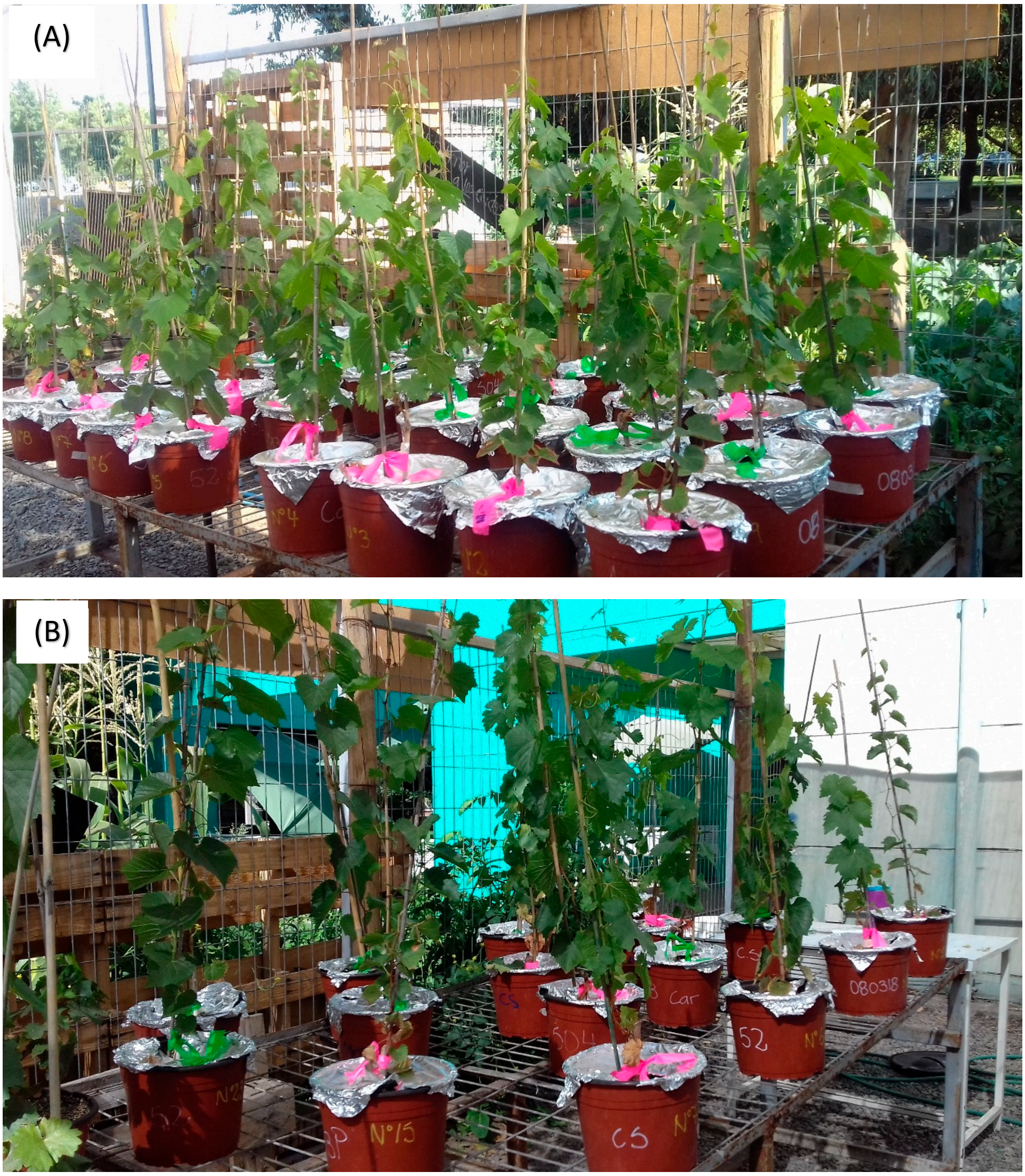

Figure S1 (A) Plants from the genotype experiment at the onset of the trial. (B) Plants at the end of the Ni treatment. Pink ribbon = Ni treatment plants; Green ribbon = Fi treatment.

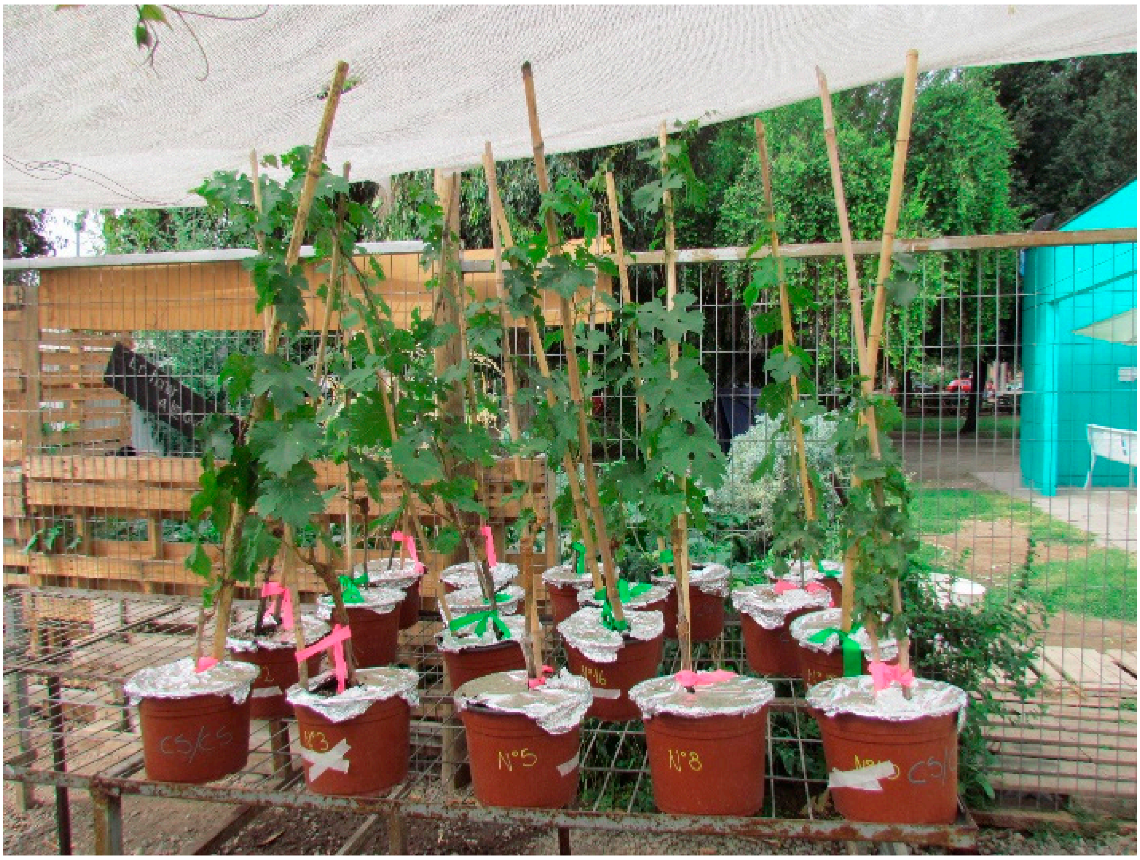

Figure S2. Plants from the scion/rootstock combination experiment at the end of the Ni irrigation treatment (Day 14). Pink ribbon = Ni treatment plants; Green ribbon = Fi treatment.
